# Breaking barriers: gender diversity and environmental identity in Pakistan's ranger workforce

**DOI:** 10.3389/fsoc.2025.1575590

**Published:** 2025-09-24

**Authors:** Hamera Aisha, Rizwana Aziz, Faiza Sharif, Britt Thielen, Rohit Singh, Haseena Anbarin, Fareeha Naseem, Rabia Tahir

**Affiliations:** ^1^World Wide Fund for Nature-Pakistan (WWF-Pakistan), Lahore, Pakistan; ^2^Punjab Wildlife and Parks Department, Govt. of Punjab, Lahore, Pakistan; ^3^Sustainable Development Study Centre (SDSC), Government College University, Lahore, Pakistan; ^4^WWF-Cambodia, Phnom Penh, Cambodia; ^5^World Wildlife Fund, Washington, DC, United States; ^6^Ministry of Climate Change & Env. Coordination, Govt. of Pakistan, Islamabad, Pakistan

**Keywords:** gender diversity, ranger workforce, gender inclusion, protected and conserved areas (PCAs), environmental psychology, identity theory, place attachment

## Abstract

Gender diversity enhances conservation outcomes by fostering inclusive decision-making and more effective policies. This study presents Pakistan's first national, gender-disaggregated analysis of its biodiversity conservation ranger workforce, examining women's perceptions, diversity barriers, and how ranger roles shape environmental identity and place attachment,. Using a mixed-method approach, we engaged 49 female and 191 male rangers, employers, and officials from wildlife, forest, and fisheries departments across all provinces and territories. Findings reveal a stark gender imbalance, with women constituting 2.6 percent of the workforce. Female rangers reported stronger biodiversity protection motivation and environmental identity, yet perceived far less acceptance in their departments. Key barriers included the male-dominated image of the profession, non-inclusive policies, recruitment biases, social and family restrictions, absence of female role models, and safety concerns. Despite their strong commitment, women disproportionately face inadequate resources, ill-fitting equipment, safety risks, and work-life balance challenges. Addressing these systemic barriers requires embedding gender equity in recruitment, promotion, and training. Workplace policies must ensure proper equipment, safety, and accommodations, while fostering an enabling environment that recognizes and supports women's contributions. Embedding gender diversity in conservation is essential for building a resilient and sustainable ranger workforce capable of delivering biodiversity and climate goals.

## 1 Introduction

Gender diversity enhances the effectiveness of conservation organizations and their operational teams. Involving women in conservation has been shown to improve outcomes (Elligson et al., 2023; James et al., 2021). “Gender” refers to socially constructed roles and traits associated with femininity and masculinity, and these can vary across societies and can change over time (Zweifel, 1997). Women and men contribute distinct knowledge, perspectives, and skills to biodiversity conservation (Elligson et al., 2023; James et al., 2021; Singh et al., 2020; UNDP, 2024). Yet, women comprise only 3–11 per cent of the global ranger workforce, with some countries reporting no female rangers in the workforce (Seager et al., 2021; Belecky et al., 2019; Singh et al., 2020). Achieving gender equity is critical for inclusive policies and effective decision-making, as emphasized in Target 3 of the Kunming-Montreal Biodiversity Framework (International Ranger Federation, 2021, 2019; Biermayr-Jenzano, 2003; Galliers et al., 2022; Graham et al., 2021).

Despite its benefits, gender diversity in conservation remains limited, with the profession historically shaped by a male-dominated “macho” culture. This culture often leads to limited acceptance of women, occupational segregation into “gender-appropriate” roles, and exclusion from decision-making processes (Prokos and Padavic, 2002; Veldman et al., 2017; Bishu et al., 2021; Christie and Giri, 2011). Women often face exclusion from critical decision-making processes and are steered toward administrative roles, limiting their impact (Belecky et al., 2019; Moreto et al., 2019; Seager et al., 2021). The physically demanding nature of ranger work, combined with challenges in balancing professional and family responsibilities, further limits women's participation (Elligson et al., 2023; Christie and Giri, 2011).

Despite these barriers, female rangers contribute valuable strengths, including effective communication, community engagement, etc. alongside the core competencies required of all rangers (Seager et al., 2021). The Hyeres Ranger Declarations from the 10th World Ranger Congress call for safe and enabling environments for women, emphasizing gender inclusivity. Similarly, the Global Ranger Perception Survey identifies persistent barriers such as underrepresentation in leadership and inadequate workplace policies underscoring the need to address these gaps and promote greater gender diversity worldwide (Elligson et al., 2023; Seager et al., 2021).

Beyond policy gaps, institutional or cultural barriers, an often-overlooked aspect of gender inclusion in conservation is the psychological dimension, specifically how individuals connect to their work and the environments they protect. Person–environment fit theory suggests that alignment between personal values and the work environment fosters wellbeing, motivation, and retention (Wacker et al., 2021). Environmental identity theory suggests that individuals who view their relationship with the natural world as central to their self-concept are more likely to adopt pro-environmental attitudes and behaviors (Clayton, 2003). Furthermore, place attachment theory highlights how emotional and psychological bonds to specific places can influence conservation commitment and stewardship behaviors (Scannell and Gifford, 2010; Mook et al., 2022). For women rangers working in male-dominated contexts, the absence of inclusive policies or acceptance may disrupt these psychological alignments and long-term presence in the field.

Despite international momentum to improve gender equity in conservation, female participation in Pakistan's ranger workforce remains extremely low, often limited to administrative or non-operational roles. There is a need for more country-specific studies to better understand the contributions and challenges of women rangers, allowing for informed policy-making and effective conservation planning (Seager, 2021).

In Pakistan, women make up only 1 per cent of the wildlife ranger workforce. A 2022 preliminary study with 23 rangers identified barriers such as limited training access, restrictive gender norms, and systemic exclusion per cent. The study highlighted the need for a national-scale assessment to better understand gender inclusiveness, enabling working conditions and perceptions about the female rangers, and to develop actionable strategies to address prevailing inequities (Hamera et al., 2023). While gender dynamics in ranger workforces have been studied in Africa, Latin America, and parts of Asia, no systematic, empirical assessment exists for Pakistan. This study fills that gap through the first gender-disaggregated analysis of ranger roles, workplace experiences, and institutional barriers across multiple provinces and territories

This research builds on the 2022 preliminary assessment, this national-scale study spans wildlife, forest, and fisheries departments. It explores the perceptions and attitudes of male and female rangers regarding the representation of women in the ranger sector, the workplace culture, and the barriers women face in a predominantly male-dominated field. The research is structured in two segments. Firstly, it examines the perspectives of men and women rangers regarding their jobs and the enabling factors within their work environment. Secondly, based on the ranger perceptions, it identifies the barriers and factors limiting the inclusion of women in the ranger workforce, such as general acceptance, social norms and gender expectations, and institution-specific deficiencies like career growth opportunities, workplace safety and harassment. Our study also integrates concepts from environmental psychology to explore how ranger roles shape women's environmental identity, emotional connection to nature, and relationships with the communities they serve. These psychological dimensions are crucial for understanding both workplace inclusion and the effectiveness of conservation engagement yet remain underexplored in ranger-focused research.

## 2 Methodology

### 2.1 Study area

The study spans all of Pakistan, covering rangers, employers, and senior officials from wildlife, forest, and fisheries departments across all provinces (Punjab, Sindh, Khyber Pakhtunkhwa, Balochistan) and territories (AJK, Gilgit-Baltistan, Islamabad). The study was carried out between February–October 2024. For consistency, the term “ranger” encompassed all frontline staff involved in biodiversity conservation in the field.

### 2.2 Sampling approach

A purposive sampling strategy was adopted to ensure representation across all provinces and territories, ranger roles, and genders. The sample was designed to be broadly representative in coverage by including participants from every geographic region and ranger category within the wildlife, forest, and fisheries sectors. Given the small number of women rangers in Pakistan and varying departmental access, the study sought to include as many women rangers as possible, supplemented by male rangers, employers, and senior officials to capture a broad range of perspectives. Snowball sampling was also used for connecting with additional respondents through referrals, especially in remote areas.

### 2.3 Data collection tool

The data was gathered using a mixed-method approach, combining surveys (with closed and open-ended questions), interviews, and focus groups discussions. The data collection tools were reviewed by experts from the World Wide Fund for Nature (WWF), the International Ranger Federation, academia, and a gender specialist. The survey tool was approved by the Ethical Review Board at the Government College University, Lahore. The survey tools were translated into Urdu, the national language of Pakistan, and into selected regional languages where needed to ensure accessibility. All the data collection was carried out in Urdu and regional languages (*Sindhi* and *Saraiki*).

### 2.4 Collating gender-disaggregated ranger statistics

The survey also included collation of gender-disaggregated statistics of rangers. A template was developed and shared with all relevant departments along with a formal letter addressed to departmental heads. Meetings and subsequent sessions were arranged to explain the scope of the study and to collect available departmental data.

### 2.5 Data collection process

Data was gathered through in-person and online methods, ensuring confidentiality. Participants were briefed on objectives, confidentiality protocols, and key terms like gender, inclusion, and safeguarding. Informed consent was obtained from all participants.

Interviews and discussions with women were conducted exclusively by female members of the research team. Men were not present during interviews or discussions with women, and women were not questioned in the presence of their supervisors. All interviews with women were conducted by the women data collection team members. Surveys and interviews averaged for one hour. As the study involved government-employed rangers, letters were sent to departmental heads outlining the study's scope and seeking their support and permission to contact participants. Before each interview, participants were briefed on the study's objectives and confidentiality protocols. Three women-specific consultations and one national-level consultation were conducted to present preliminary findings, develop key recommendations, and identify areas for future research.

### 2.6 Data analysis

Data from focus group discussions (FGDs) and qualitative survey questions were analysed thematically using NVivo 12 to identify patterns and extract key themes. Codes were developed inductively from the data and refined through iterative comparison. The coding process followed an inductive approach, beginning with open coding to identify key concepts, followed by grouping related codes into sub-themes and refining them into overarching thematic categories. The coding framework was guided by environmental identity and place attachment theories, helping to group narratives on self-concept as conservationists, emotional ties to work landscapes, cultural influences, challenges, and motivations for joining or staying in the ranger profession. These theoretical lenses guided both deductive code creation and the interpretation of emergent themes. The final themes identified through the coding process were: Cultural and Societal Barriers to Gender Equality, Institutional Deficiencies in Gender Mainstreaming, Gender Disparities in Career Growth and Leadership, and Workplace Safety. Quantitative data were compiled in Microsoft Excel and analysed using IBM SPSS Statistics (Version 28). Codes were then grouped into categories and overarching themes aligned with the study objectives. Quotations and key reflections were translated into English and included under relevant themes. Each respondent was assigned a unique ID, with gender noted but specific site details excluded to maintain confidentiality.

## 3 Results

The study examines the perceptions of male and female rangers, integrating both quantitative and qualitative data. It explores key aspects such as job motivation, work-life balance, women's representation, contributions, acceptance, workplace culture, and the challenges faced by women in the field.

### 3.1 Respondent's demographics

Findings are derived from responses of 49 female and 191 male rangers, with comparative per centages used for analysis. To ensure anonymity and account for the relatively small number of female respondents, the results represent rangers collectively from the forestry, wildlife, and fisheries departments. Overall, 78.8 per cent of respondents lived with their families, with similar proportions among female (77.6 per cent) and male rangers (79.1 per cent). Whereas 40.8 per cent of female rangers and 85.3 per cent of male rangers were married. This difference was statistically significant (χ^2^ = 44.20, *df* = 2, *p* < 0.001), indicating that male rangers were more likely to be married than female rangers.

### 3.2 Representation of women in the workforce in Pakistan

The staff statistics compiled from government, provincial and territorial wildlife, forest, and fisheries departments indicate a total of 28,079 staff personnel dedicated to biodiversity conservation and protection in Pakistan. The figures reflect overall staff statistics including the frontline and administrative staff serving in these departments. These departments operate across all four provinces (Punjab, Sindh, Khyber Pakhtunkhawa, Balochistan) and territories (Islamabad Capital Territory, Azad Jammu, Kashmir and Gilgit-Baltistan) in Pakistan.

**Figure 1:**
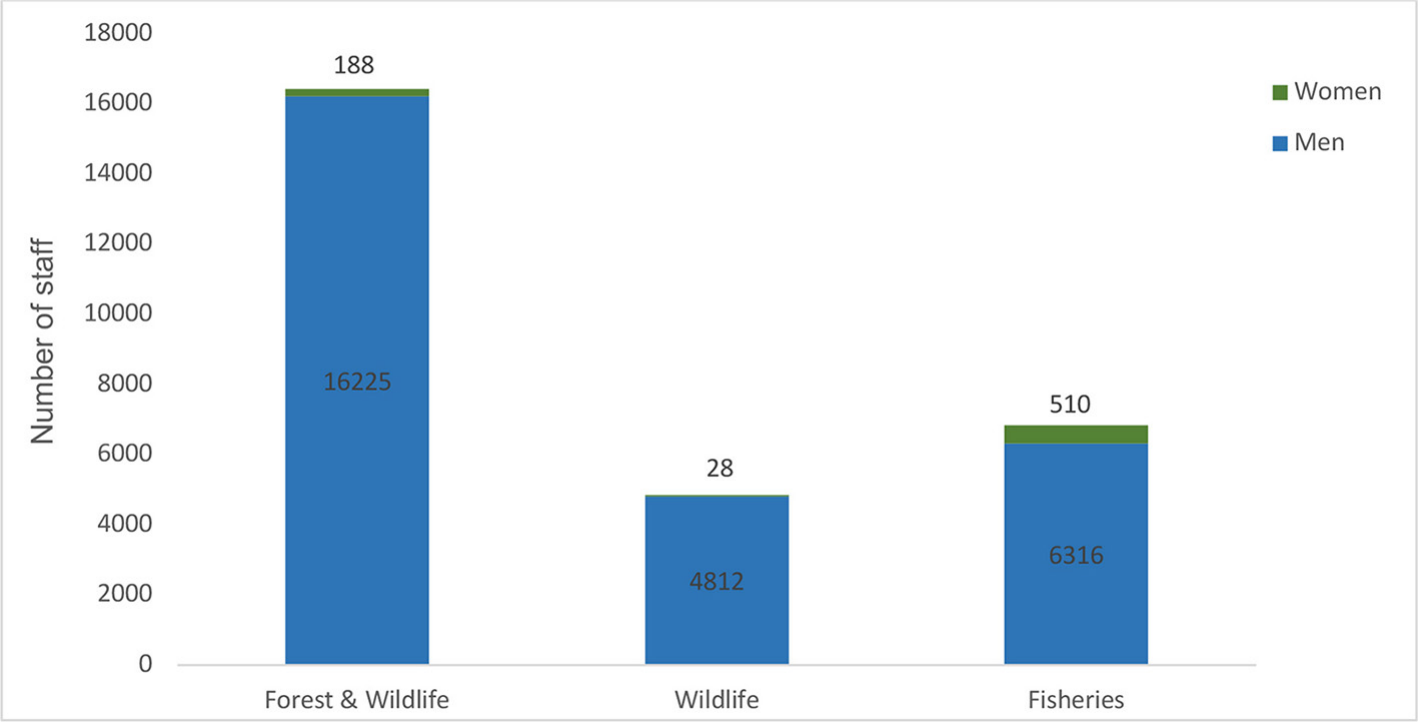
Gender-wise distribution of staff in Forest, Wildlife, and Fisheries Departments in Pakistan: workforce mandated for biodiversity protection and conservation. (Data Source: Departmental staff records collated from Wildlife, Forest, and Fisheries Departments as part of this study; Accessed July–August 2024.

In some provinces and territories, such as Gilgit-Baltistan and Balochistan, forest and wildlife departments provided combined statistics for their staff. Since most of the staff served in the forest departments, they were categorized as forest staff. In terms of workforce distribution, 58.5 per cent of the staff are employed in forest departments, followed by 24.3 per cent in fisheries departments and 17.2 per cent in wildlife departments. A stark gender imbalance is evident in the workforce composition as 27,353 (97.4 per cent) of the staff are male, while only 726 (2.6 per cent) are female (Figure 1).

The wildlife and forest departments demonstrate the lowest representation of women, with only 1 per cent female staff each, compared to 99 per cent male representation. Fisheries departments have a higher proportion of women, with 7 per cent female staff, compared to 93 per cent male. This marginally greater representation of women in fisheries departments may be partially attributed to placement of women in research and fish hatcheries projects and inclusion of daily wager staff in their personnel lists.

### 3.3 Nature conservation, a common motive for joining the ranger workforce and unique identities

We asked rangers about their motivation for joining the workforce, with a majority citing a passion for biodiversity protection and conservation. This is an expression of environmental identity, where individuals perceive themselves as part of nature and feel a moral responsibility for its care. This motivation was more common among female rangers (76 per cent) than males (60.3 per cent). According to environmental identity theory, such self-perceptions often translate into pro-environmental career choices, and our findings suggest this identity is particularly pronounced among women in the ranger workforce. When asked further, many women shared that their aspiration to become rangers was rooted in their upbringing in biodiversity rich areas, where they observed both value of nature and its degradation. They described their roles as a meaningful way to give back to the environment and contribute to its protection. These accounts reflect place attachment, the emotional and experiential bond with specific landscapes.

**Figure 2:**
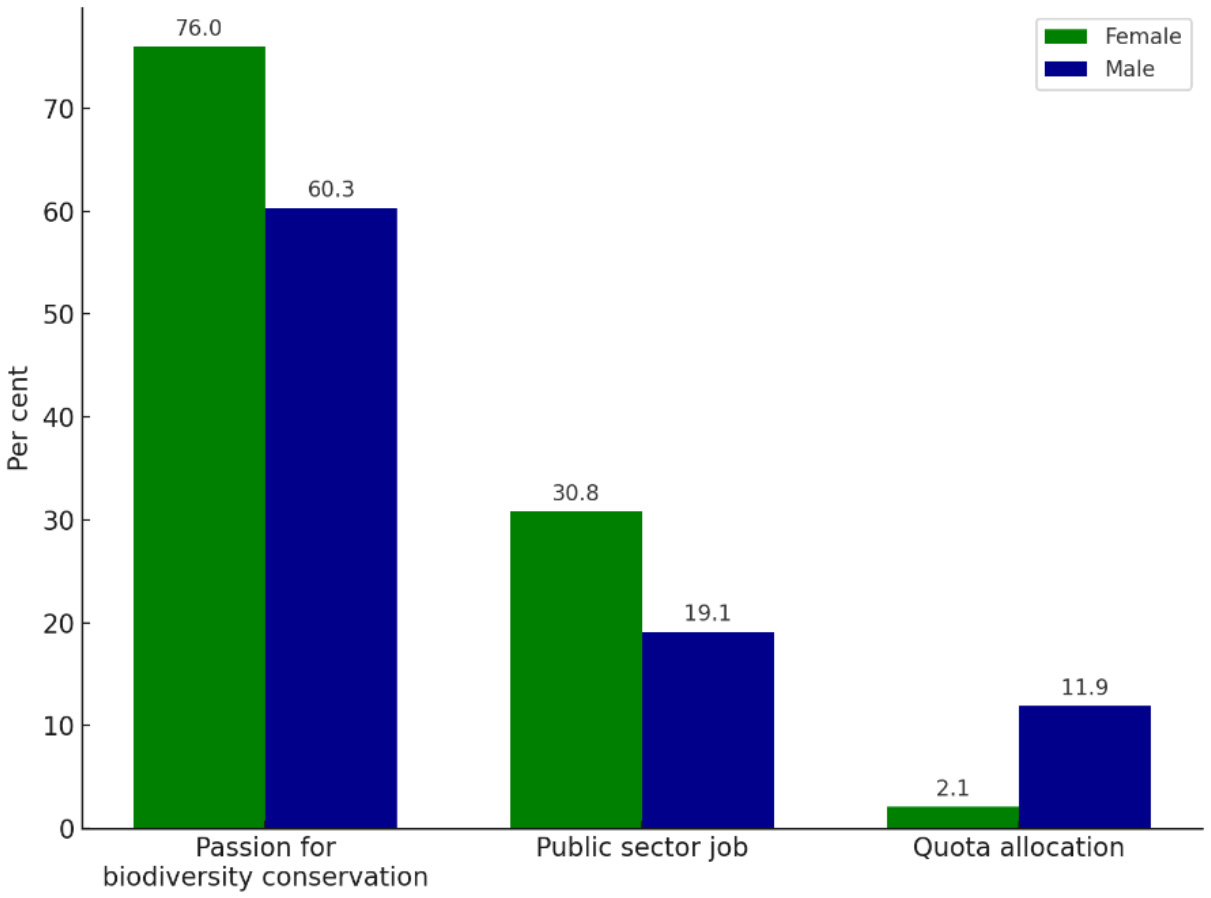
Comparison of male and female rangers' motivations for joining the workforce, showing a stronger inclination among women toward biodiversity conservation, while men are more represented in quota-based entries.

Other motives for joining the workforce were the applying to a public sector job, primarily for employment security, this had a distribution of 30.8 per cent female and 19.1 per cent male. Additionally, 2.1 per cent of female respondents entered the workforce through quota allocation, compared to 11.9 per cent of male respondents. The federal and provincial governments in Pakistan implement a quota system to support equitable representation of various regions and groups, including women and minorities, persons with disabilities, those in civil service and with provincial government positions (Figure 2).

### 3.4 Variations in years of experience between male and female rangers participated in the study

The data highlights a significant disparity in experience levels between male and female rangers (Table 1). Male respondents generally have longer service tenures, with 42.4 per cent having over 10 years of experience, demonstrating long-term commitment. In contrast, the most experienced female respondents (26.5 per cent) have 10–15 years of service, while the majority (40.8 per cent) have 3–5 years of experience, suggesting that women's inclusion in the ranger profession is recent or that retention remains a challenge. No female rangers reported over 20 years of service. A Chi-square test of independence indicated that the differences in the distribution of years of experience between male and female rangers were statistically significant (χ^2^ = 20.92, *df* = 6, *p* = 0.002).

**Table 1:** Variations in years of experience between male and female rangers, self-reported years of experience of survey respondents.

**Experience**	**Male**	**Female**
< 3 years	6.8%	10.2%
3–5 years	32.5%	40.8%
5–10 years	17.8%	22.4%
10–15 years	13.1%	26.5%
15–20 years	13.6%	0%
>20 years	15.7%	0%

### 3.5 Enabling working environment

#### 3.5.1 Enabling working environment - work-life balance

The concept of work-life balance among rangers appeared to be shaped by their unique professional experiences, cultural and social norms and personal circumstances. Both male and female respondents unanimously responded that ranger work is not a conventional 9-to-5 job, but rather a profession that demands long hours, extraordinary efforts, and unwavering dedication to fulfill its requirements.

Among male rangers, 82.2 per cent believed their organisation provides work-life balance while 11 per cent disagreed, and 6.8 per cent remained unsure. Their perception of work-life balance was often influenced by traditional gender roles, with many male rangers noting that their wives and families assume the primary burden of household and childcare responsibilities, allowing them to focus on the demands of their profession.

In contrast, 57.1 per cent of female rangers felt their job provided a reasonable balance, while 42.9 per cent disagreed. This gender difference in responses was statistically significant (χ^2^ = 12.47, *df* = 2, *p* = 0.002), indicating that male rangers were more likely than female rangers to report having work–life balance (Table 2).

**Table 2:** Perceptions of work-life balance and availability of day care facilities among male and female rangers.

**Gender**	**Support offered by their departments**	**Yes (%)**	**No (%)**	**Unsure (%)**
Male	Work-life balance	82.2	11	6.8
Female		57.1	42.9	0
Male	Day care facilities available	10.5	79.6	6.1
Female		12	77.6	6.8

Female respondents described distinct challenges in managing both professional and familial responsibilities. The role of leadership was a critical factor, with 66.1 per cent of women rangers indicating supportive supervisors alleviating pressures, whereas less accommodating management exacerbated difficulties. Work-life balance becomes a major challenge for female rangers when they are posted to distant areas without any family support (41 per cent). Additionally, the lack of flexible work arrangements further hindered women's ability to balance personal and professional obligations effectively (79.3 per cent).

*According to a respondent* “*I could never fully disengage from my professional duties. When I am invited to a family gathering or event, I usually do not dress fancy, especially during bird-hunting season, as I might receive a call at any time. Even while attending family gatherings, I stay prepared for work.” (*Respondent 01, Female)

Another experienced female ranger shared “*There were difficult times, especially when I was posted to another location without family support. Unplanned patrolling in the field and raids late at night were particularly challenging, and childcare became a nightmare as my husband was not living with us.”* (Respondent 41, Female)

#### 3.5.2 Enabling working environment - day-care and childcare facilities at workplace

When inquired about childcare support, most of both female (77.6 per cent) and male (79.6 per cent) rangers reported that no daycare facilities were available within their departments. Only 12 per cent of women and 10.5 per cent of men had access to such facilities, while 6.1 per cent of women and 6.8 per cent of men were unaware of their existence (Table 2).

Access to daycare was primarily concentrated in headquarters or district offices, where these facilities often consisted of a shared space for both women's prayers and childcare. Many female respondents noted that daycare services were a recent development, though they expressed mixed opinions on their effectiveness, citing concerns that such facilities cannot fully substitute maternal care and may impact child development.

When asked about the potential usefulness of daycare services, 79 per cent of male rangers stated that they would not use them, as they do not bring their children to work. In contrast, 100 per cent of female respondents recognised the importance of such facilities and expressed willingness to utilize them if available.

*A respondent mentioned “I also believe that childcare and joint family care cannot replace a mother's care in a child's development. I experienced this firsthand when I had to leave my daughter with house help, and she became addicted to using a mobile phone, which negatively affected her development. It made me feel like I had failed as a mother.”* (Respondent 19, Female)

### 3.6 Perception of women rangers

#### 3.6.1 Perception of women rangers - acceptance of women rangers in the workforce

When asked about the acceptance of women in the workforce compared to the past, 82.2 per cent of male rangers felt there has been significant progress with more acceptance and noticeable improvements taking place with regards to gender inclusion within their departments.

“*In the past we used to have more conservative seniors and staff. Things are changing now; we are witnessing more women coming to work in our departments and many of them are very competent.”* (Respondent 102, Male)

However, this perspective was not entirely shared by female rangers. Only 49 per cent of female respondents agreed that substantial progress has been made, suggesting significant differences (χ^2^ = 12.01, *df* = 1, *p* < 0.001). Women participants emphasised the crucial role of leadership and departmental culture in shaping the behavior of male colleagues.

A female ranger further added “*I don't believe men truly accept us. For me, dealing with administrative and clerical staff in the department has always been a significant challenge, especially as a woman. They are often unsupportive and require me to go through a cycle of struggles or wait for direct orders from my supervisor to address my requests”. (Respondent 31, Female)*

Some positive examples emerged, demonstrating growing support from male colleagues and leaders in fostering an inclusive work environment.

“*My supervisors and colleagues are very respectful. For instance, when we return from the field and resources such as vehicles are limited, they always offer me the front seat while four of them fit in the back. It would have been an easy option for them to ask me to stay back but we managed our field together as a team. As the only senior female ranger, I am frequently given the opportunity by my supervisor to represent my department at senior-level forums.” (Respondent 37, Female)*

These findings indicate an evolving but uneven landscape in the acceptance of women within the ranger workforce. While male respondents perceived substantial progress, many female rangers still face institutional and cultural barriers that hinder their full integration and professional growth.

#### 3.6.2 Perception of women rangers - do women leave their jobs more often than men?

**Figure 3:**
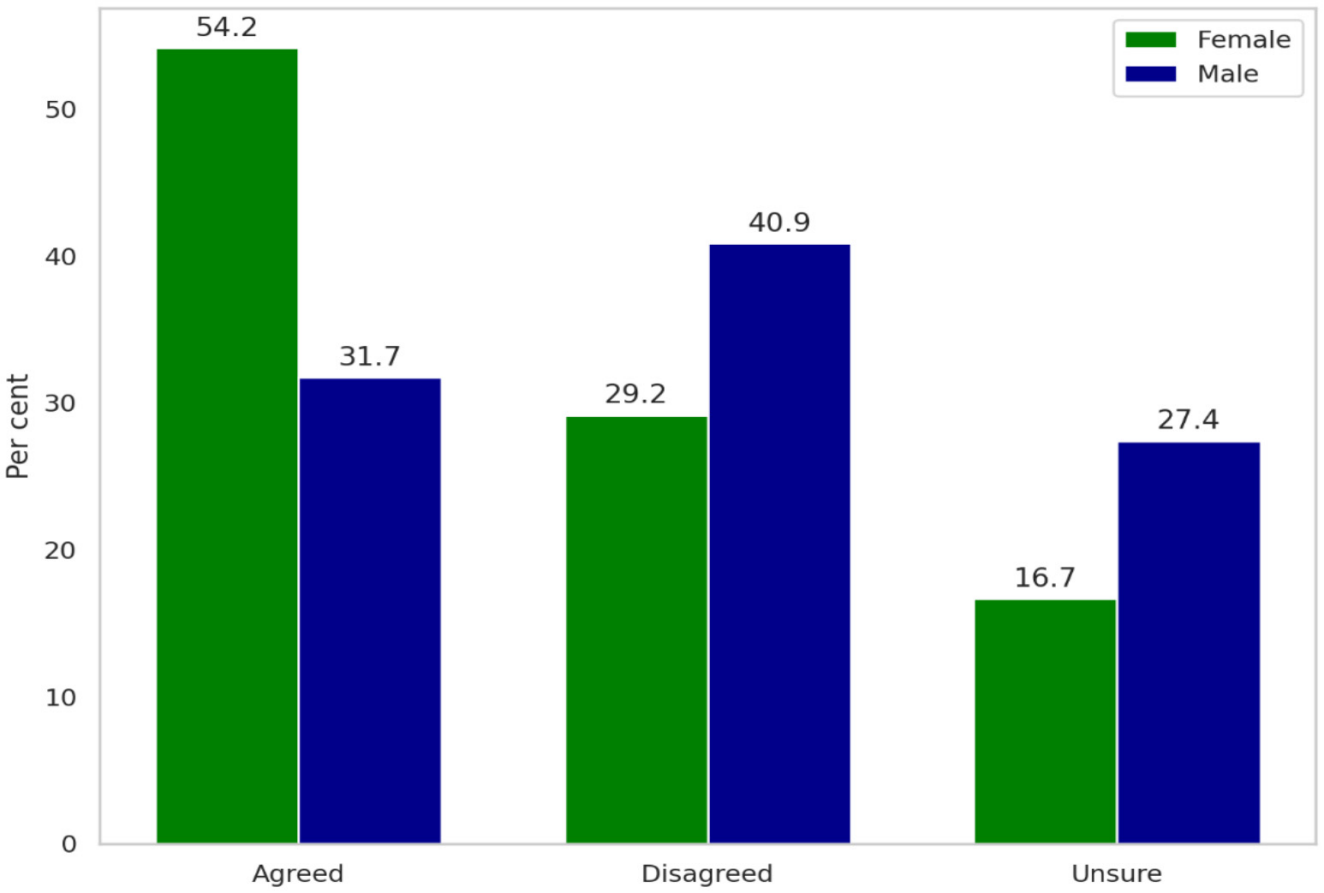
Comparison of male and female rangers' perceptions on whether women leave the ranger workforce at higher rates than men, highlighting differences in agreement, disagreement, and uncertainty.

When asked whether turnover rate is higher among women in comparison to their male counterparts, 54.2 per cent of women agreed, 29.2 per cent disagreed, and 16.7 per cent were unsure. Among men, 31.7 per cent believed women had a higher turnover rate, while 40.9 per cent disagreed and 27.4 per cent were unsure (Figure 3).

This variation in perspective suggests a potential gap in awareness or concern regarding women's representation and retention in the ranger workforce. Female rangers' lower perception of inclusion might relate to weaker institutional attachment if their experiences in the workplace are less supportive. Notably, this finding contrasts with the previously expressed male perception that women are increasingly accepted and that positive changes are occurring within the department.

#### 3.6.3 Perception of women rangers - reasons that limited women apply to the ranger jobs

Both male and female respondents identified six key factors that make the ranger profession less appealing to women. A male-dominated image was the most cited barrier, reported by 44.9 per cent of women and 53.4 per cent of men. Lack of gender inclusive policies and guidelines was highlighted by 26.5 per cent of women and 9.9 per cent of men, while the absence of female role models was noted by 6.1 per cent of women and 3.7 per cent of men.

Biases in recruitment processes were reported by 8.2 per cent of women and 7.9 per cent of men, while social and family restrictions were identified by 14.3 per cent of women and 14.1 per cent of men. Additionally, safety concerns were mentioned by 11 per cent of male respondents, reflecting broader concerns about security in the field (Table 3).

**Table 3:** Barriers limiting women's applications to ranger jobs as reported by male and female respondents (per cent).

**Barrier**	**Female (%)**	**Male (%)**
Male-dominated image of the profession	44.9	53.4
Lack of gender-inclusive policies/guidelines	26.5	9.9
Social/family restrictions	14.3	14.1
Biases in recruitment processes	8.2	7.9
Absence of female role models	6.1	3.7
Safety concerns	0	11

The most cited barrier by both genders was the male-dominated image of the profession, followed by lack of gender-inclusive policies, social/family restrictions, and safety concerns.

### 3.7 Barriers to gender diversity in the ranger workforce

Discussions with both male and female rangers revealed four key themes as significant barriers to women's participation in the ranger workforce: (1) cultural and societal norms, (2) institutional deficiencies in gender mainstreaming, (3) gender disparities in career advancement, and (4) workplace safety combined with limited enabling environments (Table 4). These themes were further classified by their primary level of analysis including individual, institutional, cultural/societal, or work environment. Harassment was excluded from this framework to maintain focus on broader systemic factors affecting women's inclusion and retention in the ranger workforce.

**Table 4:** Theme-wise list of the factors identified by men and women rangers as key barriers that make the ranger workforce a limited opportunity profession for women in Pakistan.

**Theme**	**Level of analysis**	**Sub-themes**
Theme 1: Cultural and Societal Barriers to Gender Equality	Cultural/ Individual	Traditional gender norms; Limited family/community support for women in field roles
Theme 2: Institutional Deficiencies in Gender Mainstreaming	Institutional	Weak implementation/awareness of gender policies; Biased recruitment, promotion & postings; Inadequate enabling facilities (childcare, gear, sanitation)
Theme 3: Gender Disparities in Career Growth and Leadership	Institutional	Bias in promotions; Restricted participation in policymaking; Lack of gender-sensitive planning
Theme 4: Workplace Safety	Institutional	Field safety risks; Hostile environments; Everyday sexism; Inadequate protective resources

#### 3.7.1 Barriers to gender diversity - cultural and societal barriers to gender equality

Within the cultural/societal and individual levels of analysis, cultural and social norms were identified as significant barriers limiting women's engagement in the ranger workforce. These expectations often align with aspects of place attachment, which refers to the emotional and symbolic bonds individuals form with specific locations (Scannell and Gifford, 2010). For many women, their identity and social role are strongly tied to their home community, where they are expected to remain present, raise children, and contribute to domestic life. Ranger work often involves postings to distant areas, extended fieldwork, and working alongside male colleagues, disrupts these bonds and conflicts with culturally sanctioned roles.

“*My parents and friends are unhappy with my work. They find it strange and are concerned about me working alongside so many men. They still believe I should look for a better and more stable job such as a lectureship.” (Respondent 16, Female) &* Another women respondent added “*My colleague's father used to accompany her to the field during raids, as the family did not find it socially acceptable for her to go alone at night.” (Respondent 19, Female)*

Men also described pressures linked to place-based roles, with cultural norms reinforcing expectations that they embody strength, fearlessness, and resilience within their work environment. These norms, while rooted in social identity, can intensify stress and discourage emotional openness, making it harder to process demanding or traumatic field experiences.

#### 3.7.2 Institutional deficiencies in gender mainstreaming

Institutional deficiencies significantly limit opportunities for women in Pakistan's ranger workforce. These included weak implementation of gender-related policies, low awareness of existing guidelines, insufficient financial and organizational resources, inadequate monitoring and evaluation systems, and ineffective communication strategies for gender sensitization. Deep-rooted gender stereotypes within decision-making structures were also noted as a constraint. Postings that require women to be stationed away from their homes and families present additional challenges, particularly in a cultural context where domestic responsibilities disproportionately fall on women.

“*The Government of Pakistan has policies that supports the placement of unmarried women staff in their hometowns. However, many unmarried women in our department have been transferred to distant locations. While this may not appear to be a significant issue at first glance, the economic burden and social security concerns for women are substantial. Finding safe accommodation and managing the daily commute becomes particularly challenging, especially when government residences or rest houses are unavailable. These transfers and postings are often short-term, even though the rules stipulate that such placements should last for a minimum of two years.”* (Respondent 18, Female)

#### 3.7.3 Barriers to gender diversity - gender disparities in career growth and leadership

Gender disparities in career advancement and leadership roles remain significant challenges for women in the ranger workforce. Respondents reported that women face persistent challenges in advancing to leadership and decision-making positions in the ranger workforce. Biases in promotion and restricted participation in policy-making and consultation processes were identified, with no established mechanisms to ensure women's inclusion.

Among survey respondents, women's presence in senior roles was limited. No women reported serving as Conservator or Deputy Conservator, whereas these roles were held by three and four men, respectively. In Director positions, there were two women compared to four men, and in Assistant Conservator positions, one woman compared to five men.

Structural factors were also noted as limiting advancement opportunities. Several participants explained that promotions are determined primarily by seniority and years of service. Because women's induction into the sector is relatively recent, their chances of reaching higher leadership positions in the near term are limited.

One respondent explained, “*In our department, promotions to higher grades and leadership roles are determined by seniority and the number of years spent in service. Since the induction of women in this sector is relatively recent, it means we cannot realistically expect to reach leadership positions in the near future.” (Respondent 16, Female)*

Another issue raised was the persistence of stereotypes and distinct ideas about the roles women should take on. For example, male rangers interviewed suggested that roles such as research and desk-based positions would be more appropriate for women, as they are perceived to be easier and more comfortable. However, these respondents denied the existence of any discrimination, instead attributing the limitations women face in the sector to their own personal choices.

#### 3.7.4 Barriers to gender diversity - workplace safety

Safety is a significant concern for both male and female rangers, though women described facing heightened risks, particularly in remote field settings. Respondents reported that a hostile work environment Coupled with security challenges act as a deterrent for women considering or continuing in the profession. Women also highlighted that fieldwork demands, including long hours and unpredictable schedules, posed additional difficulties in contexts where resources and protective measures were limited.

“*Field gear is not designed for women, which means it doesn't fit our unique body structures and is often difficult to manage. The travel allowance is also insufficient to provide safer and more reliable accommodation when government-managed rest houses or facilities are unavailable during fieldwork. Male colleagues can often find a place to stay, sometimes with communities, friends, or even outdoors, but I cannot do that. How can we be expected to find accommodation and handle all the fieldwork alone?” (Respondent 21, Female)*

Inadequate access to private and clean washroom and sanitation facilities was another common concern. This lack of basic amenities increased health risks and discomfort during extended fieldwork.

“*Extended fieldwork also brings the issue of limited access to private and clean washroom and sanitation facilities. This is a basic human need, but the lack of such facilities can increase exposure to potential risks and threats in the field. There were days when I spent the entire day without using the toilet, so I avoided drinking water. However, this led to health issues for me.” (Respondent 18, Female)*

Perceptions of safety risks in remote postings were often linked to environmental identity, as some participants described their work as inseparable from their identity as protectors of particular landscapes. However, when safety measures were insufficient, this identity conflicted with personal security concerns, illustrating the tension between professional commitment to a place and the barriers that limit women's sustained engagement.

#### 3.7.5 Barriers to gender diversity -workplace harassment and everyday “sexism”

Navigating open discussions about workplace harassment proved challenging, as many respondents initially assumed the question referred specifically to sexual harassment. Most respondents either denied the existence of the issue or chose not to respond. However, among those who engaged, it became clear that both male (79.6 per cent) and female (97.9 per cent) rangers were aware of the concept of workplace harassment.

When asked about the prevalence of harassment in the ranger sector, 77.1 per cent of female rangers believed that harassment is a significant issue within the profession, while 14.6 per cent disagreed and 8.3 per cent were unsure. In contrast, male rangers exhibited more divided views; 53.5 per cent acknowledged harassment as an issue, 31.6 per cent disagreed, and 15 per cent were uncertain. This disparity suggests a potential gap in awareness or differing experiences regarding harassment in the ranger workforce.

To further understand male rangers' perceptions of harassment, particularly in office settings and fieldwork, we asked whether they had witnessed incidents, including sexist jokes. Only 9 per cent acknowledged such occurrences, while 71.7 per cent denied it, believing harassment to be a non-issue. 11.2 per cent suggested it might be happening, but lacked certainty, and 7.5 per cent were unsure. These responses highlight a gap in awareness and a reluctance to acknowledge the experiences of female rangers within the workforce.

## 4 Discussion

This study provides valuable insights into perceptions of female rangers in Pakistan, examining overall acceptance within the workforce, workplace culture, and the enabling environment, which remains insufficiently supportive for women aspiring to lead and advance in frontline conservation. We also assessed how women's inclusion in the workforce is shaped by and contributes to deeper psychological connections such as unique identities as rangers, attachment to natural places and communities, central to both personal fulfilment and conservation outcomes. However, certain limitations exist, as it was based on the perceptions of rangers working in various roles across Pakistan. It is possible that some positive reflections or reluctance to acknowledge persistent issues stem from resistance to accepting these challenges, shaped by entrenched patriarchal norms or fear of repercussions. Nevertheless, the research team employed ethical and methodological practices to encourage honest responses, ensuring that the findings provide valuable insights into the gender dynamics and challenges within the ranger workforce.

While barriers to women's participation in ranger roles are documented globally, this study situates them within Pakistan's distinctive socio-cultural and institutional context. The ranger workforce operates under a decentralized framework of provincial and territorial forest, wildlife, and fisheries departments, each with unique policies, service rules, and gender provisions. This fragmented governance, combined with entrenched patriarchal norms, region-specific security risks, and chronic resource constraints, has resulted in women making up only 2.6 per cent of Pakistan's frontline conservation workforce. This sector-specific underrepresentation reflects Pakistan's broader gender landscape, where despite constitutional commitments and recent policy measures, structural and socio-cultural barriers including restrictive social expectations and gender-based violence continue to curtail women's economic participation. Pakistan ranks 145 out of 146 countries on the Global Gender Gap Index 2022 and 161 out of 192 on the Human Development Index 2022, with women's overall labor force participation rate standing at 21 per cent, far below the global average of 39 per cent (UN Women, 2023).

This extremely low representation also mirrors global trends, per cent, where women constitute approximately 3-11 per cent of the ranger sector (Belecky et al., 2019; Singh et al., 2020; Seager et al., 2021) and parallels broader gender gaps in STEM fields (UNESCO, 2024; Marques et al., 2024). These patterns highlight ongoing systemic barriers to women's full participation and advancement in conservation and related professions. Addressing this imbalance requires not only removing structural barriers but also ensuring that women are not expected to navigate conflicting work and family roles without institutional support. Inclusive policies and workplace recognition are essential to making the ranger profession a truly equal opportunity field (Seager et al., 2021).

The disparity in years of experience between male and female rangers respondents, coupled with the absence of women in leadership positions, appears to be a critical factor influencing the underrepresentation of women in the ranger profession. This lack of representation, particularly the absence of female role models, makes the profession less appealing to young women. This issue can be partially explained by the leaky pipeline phenomenon, which describes the progressive decline in the number of women advancing from early career roles to senior positions (Resmini, 2016). These trends are often shaped by implicit biases and societal expectations that steer women toward office-based or perceived ‘safer' roles (Marques et al., 2024; Seager, 2021). Combined with unsupportive departmental attitudes, these pressures may contribute to higher attrition rates among women in the ranger workforce. Future research could focus on gender-disaggregated trends, examining factors influencing women's decisions to leave the profession and the structural barriers that hinder their career advancement.

While resource and equipment deficiencies affect both male and female rangers, women are disproportionately impacted. The absence of properly fitted uniforms, designed primarily for men, and the lack of adequate facilities, such as restrooms, create additional barriers for female rangers (Seager, 2021). Women respondents in the study identified challenges unique to their experiences, including ill-fitting field uniforms and increased safety risks arising from the absence of adequate accommodations in remote field settings. These factors exacerbate the difficulties faced by women, who may also be at a heightened risk of harassment and violations. Governments bear the primary responsibility for ensuring adequate support for rangers and must take decisive action to improve working conditions and ensure access to essential resources (Bossert et al., 2024; Elligson et al., 2023; Seager, 2021).

Women rangers in this study expressed a strong commitment to nature conservation, often rooted in early exposure to biodiverse landscapes in their home regions. For many, becoming a ranger not only reinforced this commitment but also shaped a renewed sense of identity both as guardians of nature and as pioneers in their communities, with some being the first women in their departments or families to take on the role. Despite systemic barriers and challenging working conditions, they described their work as meaningful, fulfilling, and a source of pride, particularly in protecting species they had once seen in decline. This identity attachment aligns with environmental identity theory, which suggests that central identities guide behaviour and bring meaning (Stryker and Burke, 2000), and with place attachment theory, which highlights how emotional bonds with natural environments strengthen conservation commitment (Scannell and Gifford, 2010). These findings point to the need for workplace environments that support women's lived realities and relational leadership styles, as a strong person–environment fit improves job satisfaction and reduces turnover (Caplan, 1987). Future research should further examine how gender inclusion influences environmental identity development, emotional resilience, and well-being (Lawrence et al., 2025).

Women rangers participated in this study also reported unique experiences of community trust and engagement. One such example came from a ranger working on Indian pangolin rescue, when communities rescued pangolins from poachers, they would wait for her rather than handing the animal to male rangers citing trust in her compassion and intentions. Another participant shared that her presence made communities more approachable and cooperative, especially in challenging sites. She attributed this to both cultural norms and the respect she had earned by working in a traditionally male-dominated field. These experiences suggest that women's inclusion in conservation not only improves community relations but also fosters important psychological outcomes such as increased environmental identity, emotional connection to nature, and a stronger sense of place, all well-established in environmental psychology (Clayton, 2003; Scannell and Gifford, 2010). Their ability to foster trust, demonstrate care-based leadership, and form deep personal attachments to the natural world reflects how gender diversity enhances the emotional and relational dimensions of environmental protection.

Improving gender equity and equality in Pakistan's ranger workforce requires both operational reforms and broader institutional change. This will also require dismantling barriers that confine women to “gender-appropriate” roles, such as administrative or secretarial work, and ensuring equal access to field, operational, and leadership positions (Christie and Giri, 2011; Seager, 2021). Recruitment and promotion processes must be merit-based, with targeted training, structured leadership mentoring, and ongoing capacity-building opportunities, as access to professional development and guided career support has been shown to be critical to job satisfaction, retention, and advancement in male-dominated professions (Todak et al., 2021; Cordner and Cordner, 2011). Cultural barriers, particularly the persistence of a machismo culture, mirroring patterns in policing where discriminatory norms hinder advancement (Rabe-Hemp, 2009; Archbold and Schulz, 2012). Addressing these issues requires gender-sensitivity training, robust anti-harassment policies, and institutional reforms that provide flexible scheduling, childcare support, and appropriately designed uniforms and safety equipment, especially given the challenges women face in balancing family obligations with operational demands (Cordner and Cordner, 2011; Kurtz et al., 2012).

Building on these findings, we recommend a multi-level strategy to advance gender equity in Pakistan's ranger workforce. At the institutional level, gender equity should be embedded into recruitment, promotion, and training frameworks, alongside provisions for fitted uniforms, safe accommodations, and anti-harassment protocols, in line with the International Ranger Federation's principles on equity, equality, and rights in the ranger workforce (International Ranger Federation, 2024). The wildlife, forest, and fisheries regulations should undergo a comprehensive gender review to identify structural and legislative barriers. At the community level, the operational strengths of female rangers such as conflict de-escalation, community relations, research, and education should be actively leveraged in strategic planning and publicly recognised to inspire broader participation.

Additionally, future research should explore the institutional benefits of gender diversity within the ranger sector. Greater representation of women in leadership and operational roles has been linked to stronger organizational performance, improved decision-making, and more inclusive workplace cultures (Lebon et al., 2024). Targeted mentorship, leadership training, and flexible work arrangements are essential for retaining and advancing women in this profession, consistent with research highlighting women's interpersonal skills in enforcement contexts (Seager, 2021). These findings underscore the importance of integrating gender-sensitive policies in conservation institutions not only for ethical and social equity, but also for organizational performance. Recognizing and supporting the psychological benefits of women's inclusion, such as increased well-being, social trust, and empowerment, is essential for building a more resilient and sustainable conservation workforce (Van Oosten et al., 2017; Westerman, 2021).

## 5 Limitations

While this study offers valuable baseline insights into the gendered dimensions of ranger roles in Pakistan, it is important to acknowledge certain limitations certain limitations. Given the sensitive nature of the topic in a predominantly patriarchal context, social desirability bias may have influenced some participants particularly male respondents to present overly positive views about women's inclusion or to downplay persistent challenges (Ahmadi, 2023). Although trust-building measures, confidentiality assurances, and trained interviewers were employed to encourage openness, the influence of cultural and social norms cannot be entirely ruled out (Bergen and Labonté, 2020). Additionally, the limited number of female rangers across provinces and territories restricted our ability to conduct regional or institutional comparisons without risking the identification of individuals. While such analysis could have provided further insight into context-specific barriers, the small sample sizes might have risked compromising individual identities.

## Data Availability

The raw data supporting these conclusions of this article will be made available by the corresponding author on reasonable request.
